# Peer-Led Versus Conventional Teacher-Led Methodological Research Education Sessions: An Initiative to Improve Medical Education Research Teaching

**DOI:** 10.1007/s40670-023-01818-8

**Published:** 2023-06-15

**Authors:** Maria Anna Bantounou, Niraj Kumar

**Affiliations:** 1 School of Medicine, University of Aberdeen, Aberdeen, UK; 2 National Medical Research Association (NMRA), London, UK

**Keywords:** Peer-led teaching, Research education, Medical education

## Abstract

**Introduction:**

To enhance doctors’ engagement with research, the National Medical Research Association (NMRA) developed a research teaching series, delivering peer-led (PL) sessions by medical students and conventional teacher-led (CL) sessions by licenced physicians/lecturers. We assessed the effectiveness of the series and compared the PL and CL approaches.

**Methods:**

The teaching sessions were delivered virtually via Zoom weekly either PL or CL. Feedback was provided by participants on completion of every session using a 10-point Likert scale assessing their knowledge pre- and post-training.

**Results:**

A total of 87 participants were included generating 782 feedback forms, 367 (47.1%) for PL and 412 for CL sessions. The median knowledge scores significantly increased following each session (*p*-value < 0.05) independent of teaching approach. An overall improvement in the median knowledge score from all sessions from 5/10 to 8/10 was reported. There was no significant difference between knowledge gained from the CL or PL teaching.

**Conclusion:**

Didactic PL research training sessions are equally effective as CL sessions.

## Introduction

Practicing physicians are increasingly required to bridge the gap between clinical medicine and scientific discovery. Contemporaneous critical appraisal of many rapidly evolving and desperate interests is required to maximize patient outcomes to minimize morbidity. Research skills have become essential to the practice of modern medicine. It is reported that 37% of all Physiology Nobel prize winners are physician scientists [1]. A trend is reported of fewer physicians integrating research into their clinical careers [1, 2]. Concerns now exist about the non-academic clinician’s literacy in research [3] prompting the need to provide medical students with formal research training.

Exposure to research in the early years of medical school has been shown to improve research aptitude and awareness of academic career pathways [4, 5]. Research opportunities for medical students are aften reported to be limited with obstacles to successful engagement [5, 6]. This lack of research training and funding for research is further compounded by time constraints on a demanding syllabus [4, 5]. Identifying mentors with appropriate research opportunities is not without challenges [2]. These constraints when combined with the lack of formal training translate to concerns regarding the quality of medical student publications [7].

Advice on how to get involved in research is available and chiefly recommends utilizing university-associated opportunities [8]. Rarely does this advice address lack of funding and restricted research literacy. Combined Doctor of Medicine/Doctor of Philosophy (MD-PhD) programs have been instated to enrich the medical curriculum with protected research development [9], but many medical schools lack resource or incentive to offer these programs.

To combat this, a number of non-profit organizations have been developed providing training while promoting research to medical students. One such institution is the National Medical Research Association (NMRA). This UK-based society aims to limit the barriers students face in involvement in research. The NMRA developed a 13-session research training series providing participants with knowledge required to undertake research. Weekly sessions were delivered either conventionally by licensed physicians and university lecturers (conventionally led) or by medical students (peer-led).

Peer-led (PL) teaching has been suggested to be an appealing alternative to clinicians, with students reporting value in peer-to-peer learning [10, 11]. A benefit of this delivery lies in peers understanding the difficulties faced by students allowing for more accessible explanations. Peers may also prove to be more approachable than teaching faculty. Several formal “medical student-as-teacher” programs have been developed for provision of such training [12].

PL research training has been explored previously in the form of applied learning. A three-step mentorship program was introduced, whereby junior pharmacy students were required to undertake prespecified research-related tasks overseen and guided by senior pharmacy students, with the completed task reviewed by a mentor (pharmacist or other healthcare provider). This model resulted in overall positive outcomes [13]. While this mentorship-based peer-teaching has been efficacious, there is limited literature on didactic peer-led research teaching. Especially in the context of medical research, no literature was identified comparing the effectiveness of conventional teacher-led to peer-led didactic sessions.

Our research aims to assess the efficacy of conventional teacher-led and peer-led didactic sessions on 13 key research methodology topics, provided as part of an extracurricular weekly research training series. Efficacy of teaching was assessed using medical students’ self-reported research knowledge after attending the peer-led and conventional teacher-led training sessions.

## Methods

A thirteen-session online research education series was delivered from 04 May 2022 until 27 Jul 2022 on a weekly basis. The sessions were undertaken virtually on Zoom^Ⓡ^ (Zoom Video Communications, USA). Program advertising occurred via the NMRA social media (Facebook, Instagram, Twitter, LinkedIn). All participants were requested to complete a pre-teaching questionnaire collecting their baseline demographic characteristics. The education series was available free of charge to anyone who wished to sign up. There was no obligation to become a member of the organizing society (NMRA) at any point during the education series.

The teaching series aimed to equip participants with skills and knowledge required to conduct a research project. Each session targeted key methodological research topics. The series was developed by the members of the NMRA committee. This committee comprised junior doctors and medical students. Sessions delivered by medical students were defined as peer-led (PL), whereas sessions delivered conventionally, i.e., by licensed physicians or university lecturers, were referred to as conventionally led (CL). Speakers were chosen by the organizing members of the NMRA committee, depending on suitability, availability, and expertise. The organizing members also offered guidance to all speakers on the session’s content and delivery style, aiming to create a consistent structure for both the PL and CL sessions.

The sessions were recorded and were available for participants to review. Feedback forms were administered to all participants after the session and were also available on request at a later date. The feedback forms collected information on the self-assessed knowledge participants reported to have on every topic before and after the session, using a 10-point Likert scale. A score of 1 indicated poor knowledge and a score of 10 indicated excellent knowledge. The following research topics were covered by this education series:

1. Medical Students' Experience of Getting Involved in Research
2. Doctors' Experience of Getting Involved in Research
3. Selecting the Right Research Project
4. Quantitative research methods
5. Qualitative research methods
6. Critically appraising the literature
7. Validity, research and bias
8. Basic Quantitative data analysis
9. Research Ethics
10. Complex quantitative data analysis
11. The publication process
12. Abstract, posters and oral presentation
13. Systematic review and meta-analysis. A how to guide

A deviation in teaching plan occurred due to a recording error resulting in session 6 being delivered twice. The first session was delivered by a licensed physician (CL) and the second session by a medical student (PL), as the physician was unavailable to deliver the session a second time.

Participants who completed the baseline questionnaire, attended, and provided feedback to at least one teaching session were eligible for the study. Those that consented for their anonymous data to be used for research purposes were included in the analysis.

All items of the administered feedback forms and demographic questionnaires were designated as mandatory to confirm completeness. R project (RStudio) was employed for the data analysis. Categorical variables were reported using counts and percentages. Continuous variables were assessed using the Shapiro–Wilk test and if normal were reported as mean and standard deviation (SD) or median and interquartile range (IQR), if skewed. Likert scale data were described using median and IQR [14]. Significance was set at the following: alpha = 0.05, confidence interval (CI) at 95%, and a *p*-value ≤ 0.05.

Fisher’s exact test was used to determine independence of categorical variables. For numerical data that were normal (Shapiro–Wilk test *p*-value > 0.05), the *F*-test was used to examine the data variance, which indicated if a 2 independent sample *t*-test (equal variances, *p*-value > 0.05) or Welch’s *t*-test (*p*-value ≤ 0.05) was appropriate. If the data were skewed, an unpaired two-samples Wilcoxon test was used [15]. Finally, to determine if higher knowledge scores reported after the session were due to the sessions being teacher or peer-led, the Spearman’s rho was estimated. The teacher-led sessions were annotated as 0 and the peer-led sessions as 1. Spearman’s rho can be any value between − 1 (correlation with teacher-led method) to 1 (correlation with peer-led method).

This study was an NMRA education quality improvement project, whereby data were collected from routinely offered services and analyzed. The outcomes of this project will be used to shape future service provision, according to the Plan, Do, Study, Act (PDSA) cycle [16]. All participants included in this study consented for their data to be used for research purposes. No sensitive or medical data were collected as part of this quality improvement project and no identifiable information were retained. As such no ethical approval was gained.

## Results

A total of 132 participants signed up for the education series. From these, 87 (65.9%) fulfilled the eligibility criteria (consent, baseline questionnaire, attendance to minimum one teaching session, submission of minimum one feedback form) and were included in this study. Participant demographic characteristics are summarized in Table 1. The median age of the cohort was 21 (20–23) years old, with 55 participants (63%) being female.

**Table 1:** Demographic characteristics of included participants

Characteristic	Overall (*N* = 87)
Age (median, IQR)	21 (20–23)
Sex (*F*, %)	55, 63.2%
Y1 (*N*, %)	15, 17.2%
Y2 (*N*, %)	23, 26.4%
Y3 (*N*, %)	14, 16.1%
Y4 (*N*, %)	13, 14.9%
Y5 (*N*, %)	6, 6.9%
Y6 (*N*, %)	4, 4.6%%
Other (*N*, %)	12, 13.8%
Country (*N*, %)	
UK	42, 48.3%
India	21, 24.1%
Morocco	8, 9.2%
South Africa	6, 6.9%
Other	10 (Bahrain *N* = 1, Iran *N* = 1, Lebanon *N* = 1, Malaysia *N* = 1, Nigeria *N* = 1, Singapore *N* = 1, Somalia *N* = 1, Taiwan N = 1, Tanzania *N* = 1, Uganda *N* = 1)
Previous higher education (*N*, %)	9, 10.3% (BSc)
Ethnicity (*N*, %)	
South Asian	40, 46%
White	19, 21.8%
Arab	9, 10.4%
Black	6, 6.9%
East Asian	8, 9.2%
Mixed	4, 4.6%
Other	1, 1.2%
First-degree relatives in academia (median, IQR)	0 (0–2)
Eligible for free school meals (*Y*, %)	16, 18.4%
First-degree relatives, higher education (median, IQR)	2 (2–3)

*N* number, *IQR* interquartile range

Participant baseline research experience (Appendix 1), knowledge and confidence in undertaking research were evaluated. The results are showcased in Table 2. Baseline participant research knowledge was scored as a median of 3 (2–3) using a 5-point Likert scale. When specifically asked about different aspects of research, the median score ranged from 2 for knowledge of protocol development, statistical analysis, and publication processes to 3 for evidence hierarchy, study design, and literature review techniques.

**Table 2:** Participant baseline research knowledge and confidence

Characteristic	Research knowledge gained prior to educations series (median, IQR)	Research knowledge gained by the medical school (median, IQR)	Confidence in undertaking research independently (median, IQR)
Overall (median, IQR)	3 (2–3)	3 (2–3)	3 (2–3)
Evidence hierarchy (median, IQR)	3 (2–4)	3 (1.5–4)	3 (2–3)
Protocol development (median, IQR)	2 (1–3)	2 (1–3)	2 (1–3)
Statistical analysis (median, IQR)	2 (2–3)	2 (2–3)	2 (2–3)
Study design (median, IQR)	3 (2–3)	3 (1.75–3)	3 (2–3)
Literature review techniques (median, IQR)	3 (2–3)	3 (1.5–3)	3 (2–3.5)
Knowledge of publication process (median, IQR)	2 (1–3)	2 (1–3)	2 (1–3)

*IQR* interquartile range

Comparable scores were reported when participants were asked to rate the research knowledge gained by attending medical school on the same topics. Analogous scores were also provided by the students on their confidence in performing research independently overall and on the investigated topics. Upon evaluating the research experience of the participants, it was found that only approximately half (46, 52.9%) of the students had engaged in a supervised research project prior to the baseline assessment. Participants indicated an interest in pursuing an academic career, specifically interest in intercalation (4, 3–5), a PhD (4, 3–5), and post-graduate academic training (4, 4–5), such as the Specialised Foundation Programme in the UK, scored a median of 4 on a 5-point Likert Scale.

**Fig. 1:**
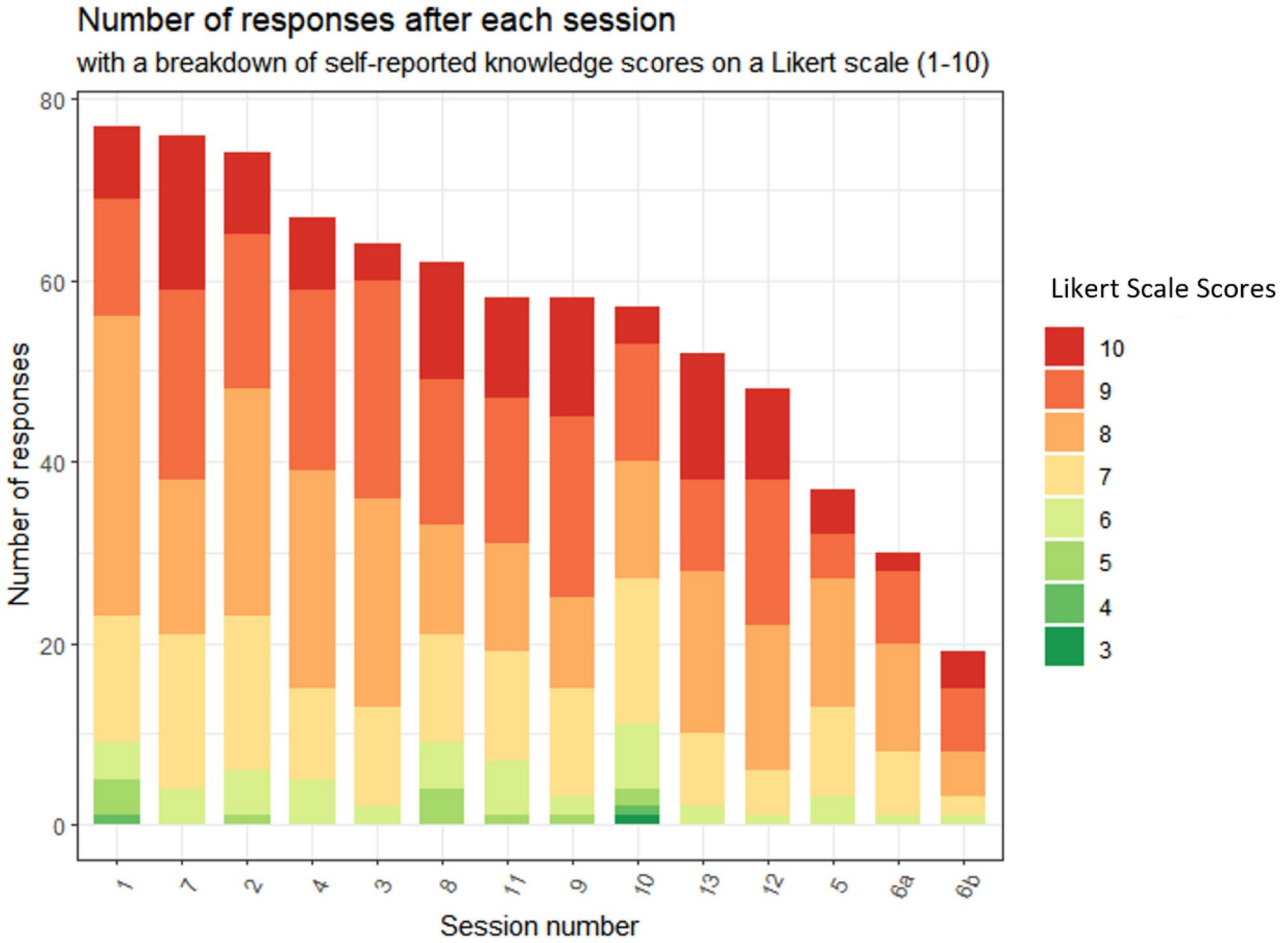
The distribution of the 782 responses across the delivered 14 sessions. Self-assessed knowledge after the session reported by the participants on a 10-point Likert scale (1, poor (dark green); 10, excellent (dark red)), counted and stacked

A total of 782 responses were recorded by 87 participants for the 14 sessions. The distribution of responses across the 14 sessions with the corresponding variability in Likert scale scores, used to describe the knowledge on the topic taught after the session, is presented in Fig. 1. It can be observed that the first session, “Medical Students’ Experience of Getting Involved in Research,” had the most responses (*n* = 77), closely followed by the seventh session “Validity, research and bias” (*n* = 76). Both sessions were PL. The session with the lowest number of responses was the “Critically appraising the literature” session, which was delivered twice, once by a medical student (PL, *n* = 19) and once conventionally (CL, *n* = 30). Session 7, “Validity, research and bias,” received the most scores that were equal to 10, i.e., “excellent knowledge” after the session, while session 10 “Complex quantitative data analysis” received lower scores that ranged between 3 and 6, corresponding to “fair knowledge.” The median score for participants’ self-assessed knowledge improved from baseline following each session, as shown in Appendix 2. This improvement was statistically significant for all sessions.

The knowledge for all 14 delivered sessions scored a median of 5 (3–6) before and 8 (7–9) after the teaching. “The Publication Process” received the lowest reported baseline knowledge with a median of 3 (2–6), improving to a median of 8 (7–9) after the session. Knowledge after the teaching did not score lower than a median of 8 for any topic. The three topics that received the highest knowledge scores after the sessions were “Critically appraising the literature,” “Research ethics,” and “Abstract, posters and oral presentation.” These were all PL sessions. Appendix 2 presents a detailed overview of all the self-assessed knowledge scores provided by the participants before and after every session.

**Fig. 2:**
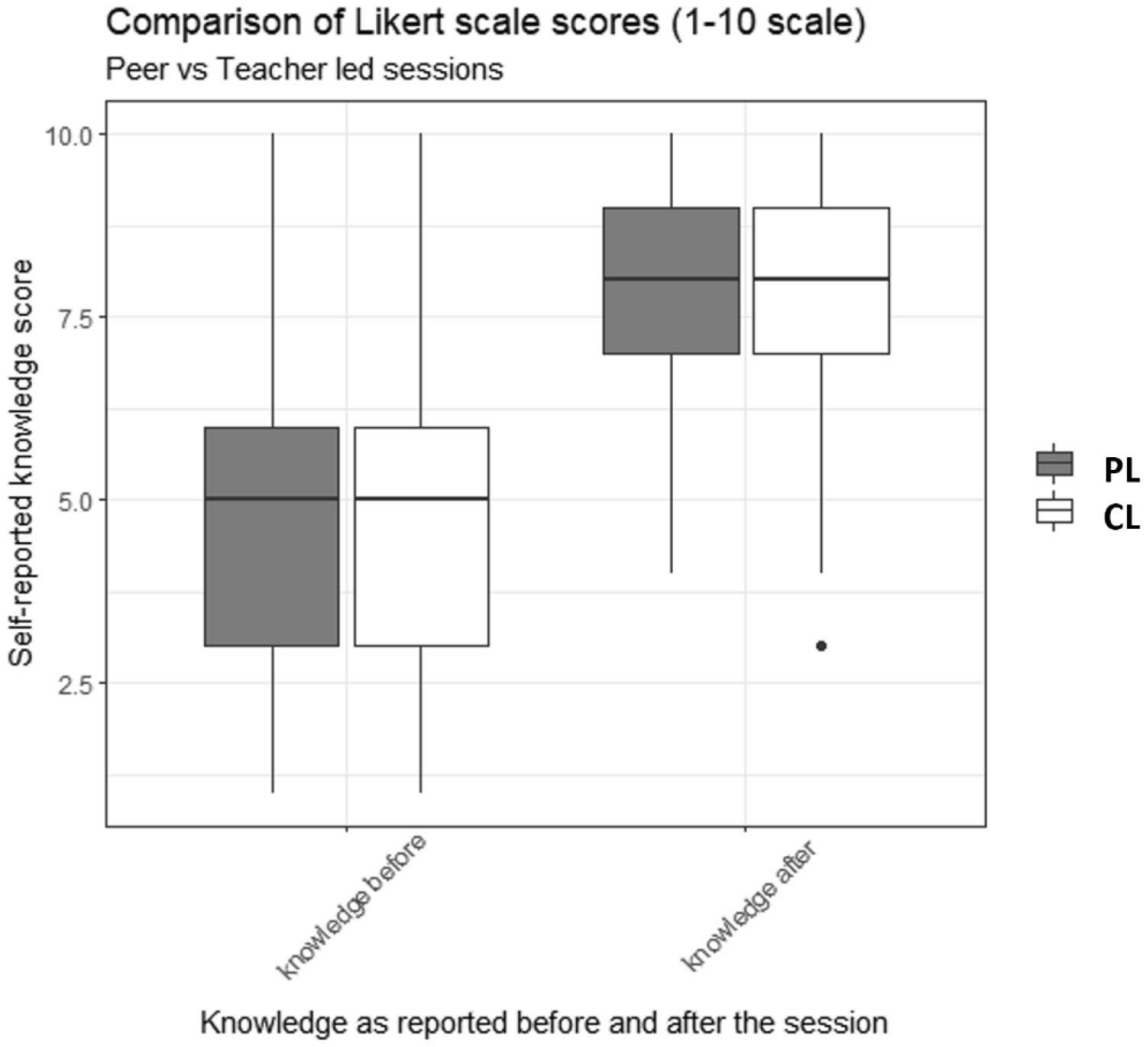
Comparison of knowledge scores between CL and PL sessions, before and after the sessions. Participant self-assessed knowledge scores reported on a 10-point Likert scale (1, poor; 10, excellent). Median and interquartile range shown. PL sessions indicated by the color gray; CL sessions indicated by the color white. PL peer-led, CL conventionally led

Further analysis was undertaken exploring if participants perceived any difference in the quality of teaching received depending on if the session was PL or CL. This was based on reported knowledge scores. The results of this analysis can be found in Table 3. From 782 submitted feedback forms, 367 (47.1%) corresponded to PL sessions, whereas the residual 412 (52.9%) to CL sessions. Variation between PL and CL session topics existed (Appendix 2). Participants’ knowledge before the teaching scored a median of 5 (3–6) for both PL and CL sessions. A median knowledge score of 8 (7–9) was reported after the teaching, for both teaching cohorts. While both the PL and CL sessions resulted in significantly (*p*-value < 0.001) improved knowledge scores after the teaching, no difference was seen when the “knowledge after” scores attributed to the CL sessions were compared to those of the PL sessions (*p*-value = 0.168). This data is recorded in Fig. 2.

**Table 3:** Comparison of knowledge scores of all CL and PL sessions, with the corresponding *p*-value. Participant self-assessed knowledge scores as reported before and after the teaching sessions, on a 10-point Likert scale (1, poor; 10, excellent)

**Characteristic**	**Responses for PL**	**Responses for CL**	*p***-value for PL vs CL**
**Number of responses (*N*, %)**	367, 47.1%	412, 52.9%	NA
**Knowledge before (median, IQR)**	5 (3–6)	5 (3–6)	0.796
**Knowledge after (median, IQR)**	8 (7–9)	8 (7–9)	0.168
*p***-value for comparison of knowledge before vs knowledge after**	< 0.001	< 0.001	NA

*N* number, *IQR* interquartile range

**Fig. 3:**
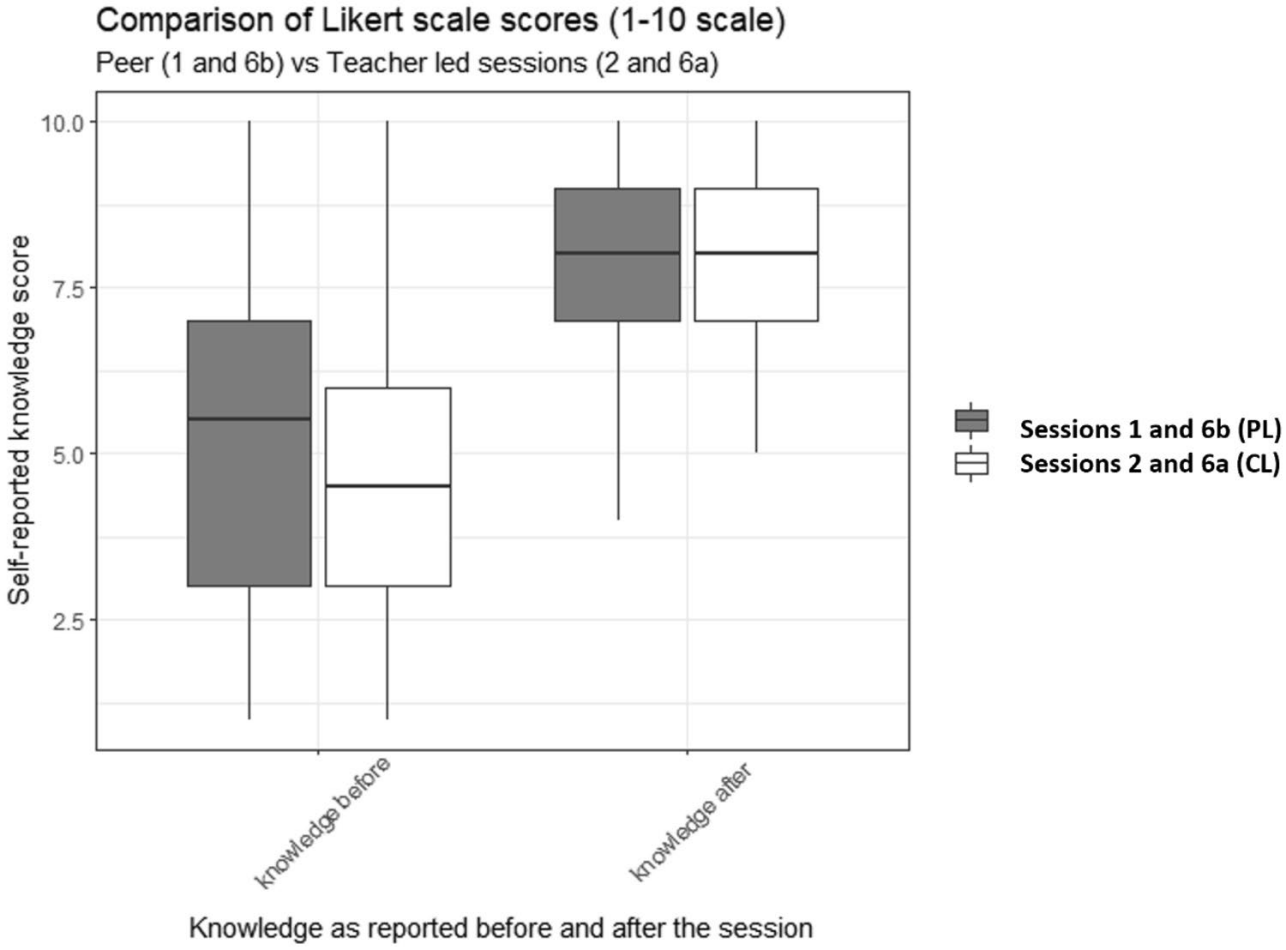
Comparison of knowledge scores between CL (2 and 6a) and PL (1 and 6b) sessions that provided teaching on the same topic. Participant self-assessed knowledge scores were reported on a 10-point Likert scale before and after the session (1, poor; 10, excellent). Median and interquartile range shown. PL sessions indicated by the color gray; CL sessions indicated by the color white. PL peer-led, CL conventionally led

The median post teaching scores were compared for sessions 1 and 2 (Getting Involved in Research) and sessions 6a and 6b (Critically appraising the literature), as they were delivered both CL and PL at separate times. This allowed for direct head-to head comparison of the two teaching approaches. No statistically significant variation in participants’ self-reported knowledge after the session was noted between the teaching arms, as presented in Fig. 3. The median knowledge score of the “Getting Involved in Research” session was similar (*p*-value = 0.517) in the PL arm (8, 7.25–9) and CL arm (8, 7–9). The “Critically appraising the literature” session also showed similar results between the two arms, with also the PL session receiving slightly higher (median = 9, 8–9) “knowledge after” scores compared to the CL session (median = 8, 7.25–9) (*p*-value = 0.096).

Finally, to further investigate the relationship between improved knowledge score and delivery of CL or PL teaching, the Spearman’s rho was estimated. Improved knowledge after the session, as indicated by the provided Likert scale scores, was correlated positively (rho = 0.0495) with PL teaching. This correlation was weak and not significant (*p*-value = 0.168).

## Discussion

This study demonstrated utility in training medical students in research methodology. Eighty-seven participants submitted 782 feedback forms with an approximately equal attendance at PL and CL teaching sessions. Just under half (42, 48.3%) of the students were attending a UK-based university, with the remainder being based in a wide variety of countries including India (21, 24.1%), Morocco (8, 9.2%), and South Africa (6, 6.9%). Most students reported to be of South Asian (40, 46%) or white (19, 21.8%) ethnicity. Considering that the organizing society was a UK-based national society, the interest in this research series by students out with the UK indicates that lack of research literacy may be a multi-national issue.

Participants reported to have a median of 2 (2–3) first-degree relatives who have completed higher education. However, 16 (18.4%) participants reported to be eligible for free school meals growing up or other similar initiative. Engaging students from all socioeconomic backgrounds with research is crucial to avoid introducing inequalities in medical education and may require institutions and governing bodies worldwide to provide support, as lacking funding and the appropriate network [5] should not become a detriment to engaging with research.

All grades of medical student Years 1–5 and intercalation were represented in the study. The majority of participants were in their first (15, 17.2%), second (23, 26.4%), and third years (14, 16.1%) of medical school. Nine participants (10.3%) had previously completed a Bachelor of Science prior to commencing their medical education. No other degrees were reported. No participants were attending a graduate entry in medicine course or were intercalating. As such, prior research experience was unsurprisingly minimal across the participants (Appendix 1). This also showcases the gap in provision of adequate research training and research opportunities by current medical school curriculums worldwide.

Median knowledge scores on all research topics improved following teaching and there was no significant difference between PL or CL sessions. The median knowledge score from all sessions was 5 before and 8 after the teaching on a 10-point Likert scale. Knowledge scores after teaching did not score less than a median of 8 for any topic suggesting significant improvement. Direct comparison between the CL and PL approach was conducted for teaching sessions 1 and 2 and 6a and 6b. No significant variation was noted between CL and PL sessions either in these head-to-head delivered sessions or across the teaching series as a whole. The topics that received the highest scores after the session were “Critically appraising the literature,” “Research ethics,” and “Abstract, posters and oral presentation,” all of which were PL sessions, demonstrating the value of peer-teaching. The “publication process” scored the lowest baseline knowledge scores, highlighting the importance in this area for additional teaching within medical school curriculum. Low exposure to the concepts required for publication could be expected due to low medical student publication numbers (Appendix 1) and the cohort being early in their medical training.

Similar results have been reported previously in the literature [17–19]. A recent meta-analysis identified 44 studies that assessed peer teaching showing similar effectiveness to expert/faculty teaching on knowledge acquisition and improved skill performance, demonstrating utility in this method [18]. This built on a systematic review that suggested that peer teaching was as effective as expert teaching [17]. Furthermore, a randomized crossover trial evaluated peer-teaching for critical appraisal skills learning [20]. Here, participants attended two tutorials, one delivered by near-peer tutors and controlled against staff tutors teaching. The cohorts were then exchanged to the alternate arm for a further two sessions. Teaching delivered by the near-peer tutors was shown to be as valuable and more acceptable compared to teaching by staff tutors. Nonetheless, this study focused on only one aspect of methodological research teaching and the peers were newly qualified doctors rather than medical students. No relevant publications examining the effectiveness of didactic PL research teaching were identified by the authors in the examined literature.

There are limitations and biases to the data presented in this publication. A variety of topics were delivered during the 13 planned teaching sessions covering a wide range of research concepts. The knowledge scores of certain sessions may reflect the complexity of the material taught, rather than differences in method of teaching (PL vs CL). Topic sessions were not controlled for complexity, and this may represent bias. Session 10 “Complex quantitative data analysis” exemplifies this. It was a CL session with the introduced concepts being traditionally challenging for students to grasp and received lower “knowledge after” scores compared to other sessions. With the exception of two topics (“Getting Involved in Research,” “Critically appraising the literature”), all other sessions did not have a direct comparator. The recruited participants expressed an interest in pursuing an academic career at baseline, resulting in the introduction of self-selection bias within the sample. This limits the applicability of the derived results. Finally, every response for each session utilized in this study was assumed to be independent to allow for the statistical analysis to take place. This however was not the case as most participants undertook more than one session of the research teaching series.

Currently, there are no training prerequisites for becoming a peer teacher allowing any medical students to take on this role. This may result in poor quality of teaching, and a lack of session structure and standardization across a curriculum. Often utilized as a learning opportunity for the medical student who is teaching, there is the risk for insufficient knowledge on the taught subject by peer tutors.

The quality of teaching is often proportional to the teachers understanding of the topic. This requires insight on the topic and the teachers command of it. This can be considered a limitation to PL teaching particularly in complex topics. The associated time required to appropriately prepare and generate sessions should not be underestimated. This has the potential to impact on increasingly time-demanding curriculums and often these sessions will be developed many times by successive generations of medical students. These are limitations that require careful consideration prior to rolling out peer-led teaching session programs [21].

## Conclusions

This study included 87 participants and analyzed 782 responses demonstrating that an online extracurricular research teaching series can significantly improve the knowledge of attendees. Attendees were found to have limited prior research experience. We showed for the first time that didactic PL sessions on key research methodological topics were as effective as CL sessions. Future work on this field is needed to develop medical student comfort in research methodology. The perceptions of medical students on didactic PL teaching need to be explored allowing for the provision of teaching sessions that meet student requirements. It is also necessary to conduct further research studies that incorporate formal pre- and post-session knowledge assessments, in an attempt to mitigate bias arising from students self-assessing their own learning.

To the authors’ knowledge, this is the first study to show that didactic PL sessions on key research methodological topics were as effective as CL. We hope that this will empower student-led bodies to improve existing research training in conjunction with medical student curriculum.

## Data Availability

Anonymized data available for review on appropriate request, held by the corresponding author.

## Appendix 1

Baseline research experience, IQR; interquartile range

**Table:**

Research experience	Response
Number of poster presentations (median, IQR)	0 (0–1)
Number of oral presentations (median, IQR)	0 (0–1)
Number of first author publication (median, IQR)	0 (0–0)
Type of project undertaken so far	
Systematic review (median, IQR)	0 (0–0)
Meta-analysis (median, IQR)	0 (0–0)
Narrative review (median, IQR)	0 (0–0)
Audit (median, IQR)	0 (0–0)
Cohort study (median, IQR)	0 (0–0)
Lab work (median, IQR)	0 (0–0)
Randomized control trial (median, IQR)	0 (0–0)
Case control (median, IQR)	0 (0–0)
One piece commentary (median, IQR)	0 (0–0)

## Appendix 2

Comparison of self-reported knowledge before and after the teaching session on a 10-point Likert scale, with corresponding *p*-value. All sessions with respective number of submitted feedback forms shown

**Table:**

Session title	Number of feedback forms	Knowledge before (median, IQR)	Knowledge after (median, IQR)	PL/ CL	*p*-value
Session 1: Medical Students' Experience of Getting Involved in Research	77	5 (3–7)	8 (7–9)	PL	< 0.001
Session 2: Doctors' Experience of Getting Involved in Research	74	5 (3–7)	8 (7–9)	CL	< 0.001
Session 3: Selecting the Right Research Project	64	5 (4–6)	8 (8–9)	CL	< 0.001
Session 4: Quantitative research methods	67	5 (4–6)	8 (8–9)	CL	< 0.001
Session 5: Qualitative research methods	37	4 (2–5)	8 (7–9)	PL	< 0.001
Session 6a: Critically appraising the literature	30	4 (3–6)	8 (7.25–9)	CL	< 0.001
Session 6b: Critically appraising the literature	19	6 (3–8)	9 (8–9)	PL	< 0.001
Session 7: Validity, research and bias	76	5 (3.75–6.25)	8.5 (7–9)	PL	< 0.001
Session 8: Basic Quantitative data analysis	62	5 (3–6.75)	8 (7–9)	CL	< 0.001
Session 9: Research ethics	58	5 (4–7)	9 (7.25–9)	CL	< 0.001
Session 10: Complex quantitative data analysis	57	4 (2–6)	8 (7–9)	CL	< 0.001
Session 11: The publication process	58	3 (2–6)	8 (7–9)	PL	< 0.001
Session 12: Abstract, posters and oral presentation	48	5 (3–7)	9 (8–9)	PL	< 0.001
Session 13: Systematic review and meta-analysis. A how to guide	52	5 (4–6)	8 (8–10)	PL	< 0.001
Median overall score for all sessions	782	5 (3–6)	8 (7–9)	All	< 0.001

*IQR* interquartile range, *PL* peer-led, *CL* conventionally led
